# MC38 Tumors Induce Musculoskeletal Defects in Colorectal Cancer

**DOI:** 10.3390/ijms22031486

**Published:** 2021-02-02

**Authors:** Joshua R. Huot, Fabrizio Pin, Alyson L. Essex, Andrea Bonetto

**Affiliations:** 1 Department of Surgery, Indiana University School of Medicine, Indianapolis, IN 46202, USA; jrhuot@iu.edu; 2 Department of Anatomy, Cell Biology & Physiology, Indiana University School of Medicine, Indianapolis, IN 46202, USA; fpin@iu.edu (F.P.); alyessex@iu.edu (A.L.E.); 3 Department of Otolaryngology—Head & Neck Surgery, Indiana University School of Medicine, Indianapolis, IN 46202, USA; 4 Indiana Center for Musculoskeletal Health, Indiana University School of Medicine, Indianapolis, IN 46202, USA; 5 Simon Comprehensive Cancer Center, Indiana University School of Medicine, Indianapolis, IN 46202, USA

**Keywords:** colorectal cancer, skeletal muscle, bone, cachexia, liver metastases

## Abstract

Colorectal cancer (CRC) is a leading cause of cancer-related death, and the prevalence of CRC in young adults is on the rise, making this a largescale clinical concern. Advanced CRC patients often present with liver metastases (LM) and an increased incidence of cachexia, i.e., musculoskeletal wasting. Despite its high incidence in CRC patients, cachexia remains an unresolved issue, and animal models for the study of CRC cachexia, in particular, metastatic CRC cachexia, remain limited; therefore, we aimed to establish a new model of metastatic CRC cachexia. C57BL/6 male mice (8 weeks old) were subcutaneously (MC38) or intrasplenically injected (mMC38) with MC38 murine CRC cells to disseminate LM, while experimental controls received saline (*n* = 5–8/group). The growth of subcutaneous MC38 tumors was accompanied by a reduction in skeletal muscle mass (−16%; quadriceps muscle), plantarflexion force (−22%) and extensor digitorum longus (EDL) contractility (−20%) compared to experimental controls. Meanwhile, the formation of MC38 LM (mMC38) led to heighted reductions in skeletal muscle mass (−30%; quadriceps), plantarflexion force (−28%) and EDL contractility (−35%) compared to sham-operated controls, suggesting exacerbated cachexia associated with LM. Moreover, both MC38 and mMC38 tumor hosts demonstrated a marked loss of bone indicated by reductions in trabecular (Tb.BV/TV: −49% in MC38, and −46% in mMC38) and cortical (C.BV/TV: −12% in MC38, and −8% in mMC38) bone. Cell culture experiments revealed that MC38 tumor-derived factors directly promote myotube wasting (−18%) and STAT3 phosphorylation (+5-fold), while the pharmacologic blockade of STAT3 signaling was sufficient to preserve myotube atrophy in the presence of MC38 cells (+21%). Overall, these results reinforce the notion that the formation of LM heightens cachexia in an experimental model of CRC.

## 1. Introduction

Despite decades of improved treatment, colorectal cancer (CRC) remains a leading cause of cancer-related death worldwide, accounting for approximately 53,200 deaths in 2020 within the United States alone [1]. Worsening the issue, mortality attributed to CRC has increased 1% annually since 2008 in individuals under the age of 55, including a 0.8% increase in individuals 15–39 [1,2]. This suggests that CRC is, and will continue to be, a critical health concern for people of all ages for the foreseeable future. Of note, it is estimated that in 80% of advanced cancers, including CRC, patients will develop very debilitating musculoskeletal deficits, which are a hallmark of cachexia. This is a progressive cancer-associated disease that impedes the ability to complete daily activities of living, reduces treatment tolerance and continuation, and worsens survival in cancer patients [3,4,5,6,7,8]. Importantly, to date, there currently are no approved treatments for cancer-induced cachexia.

Impeding the identification of viable therapies to combat cachexia is the limited availability of small animal models. This is particularly true of models that mimic the advanced clinical phenotype of cancer patients and has become a focal point for our group and others attempting to generate models of advanced metastatic cancer cachexia [9,10,11,12,13]. Supporting the increased use of models mimicking advanced CRC, we have reported not only that the formation of liver metastases (LM) exacerbates skeletal muscle wasting, but also that the formation of LM causes differential signaling within skeletal muscle compared to commonly used allograft and xenograft tumor implants [9,12]. In line with exacerbated skeletal muscle wasting, we have also shown that LM causes multi-organ wasting affecting the heart, fat and bone, exceeding the losses observed in subcutaneous CRC models [9,12].

Another confounding issue with respect to CRC cachexia research is the lack of cell lines capable of forming tumors in the widely used C57BL/6 strain, the preferred background for transgenic animals. One candidate, MC38, has revealed mixed results with respect to cachexia, with literature suggesting both non-cachectic and cachectic phenotypes, thereby warranting further investigation [14,15]. Moreover, whether the formation of MC38 LM would exacerbate cachexia similar to other experimental CRC models has not been examined.

In the present study, we sought to investigate the effects of MC38 tumors on the musculoskeletal system and examine whether the formation of LM causes heightened cachexia. Here, we demonstrated that the growth of subcutaneous MC38 tumors promotes mild skeletal muscle wasting, marked bone loss and muscle weakness. Meanwhile, the formation of MC38 LM appears to exacerbate cachexia, supporting the further use of models that mimic the clinical phenotype of advanced CRC patients.

## 2. Results

### 2.1. Growth of Subcutaneous MC38 Tumors Causes Cachexia Consistent with Fat Wasting, Muscle Loss and Weakness

**Figure 1:**
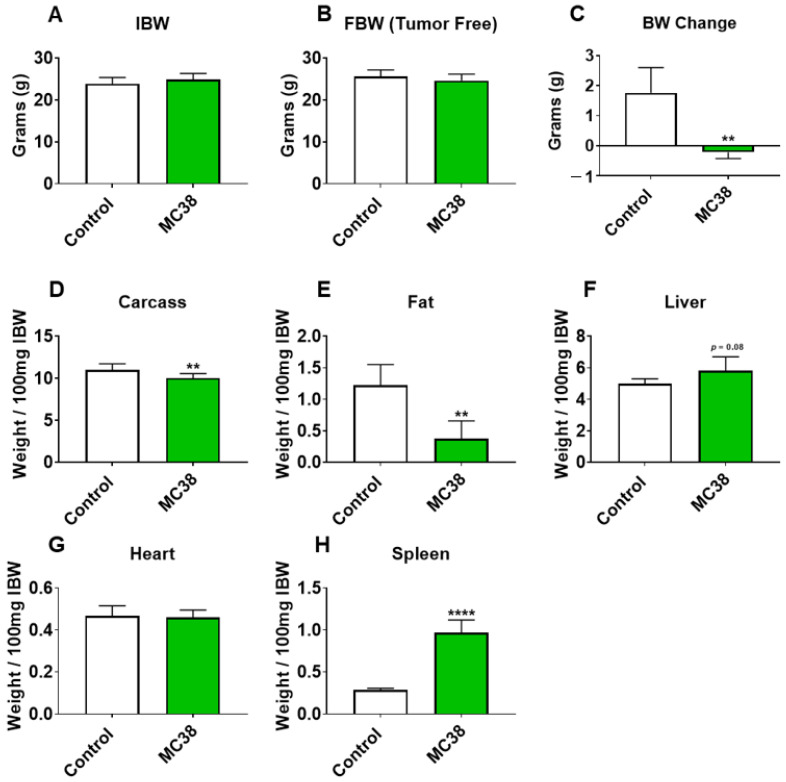
Growth of subcutaneous MC38 tumors promotes cachexia. (**A**–**D**) Initial body weight (IBW) (**A**), tumor-free final body weight (FBW) (**B**), BW change (**C**) and carcass weights (**D**) of C57BL/6 male mice (8 weeks) subcutaneously injected with MC38 tumor cells (1.0 × 10^6^ cells/mouse in sterile PBS: MC38) or an equal volume of vehicle (control) (*n* = 5). (**E**–**H**) Fat (**E**), liver (**F**), heart (**G**) and spleen (**H**) weights normalized to IBW. Data presented as mean ± s.d. ** *p* < 0.01, **** *p* < 0.0001 vs. control.

**Figure 2:**
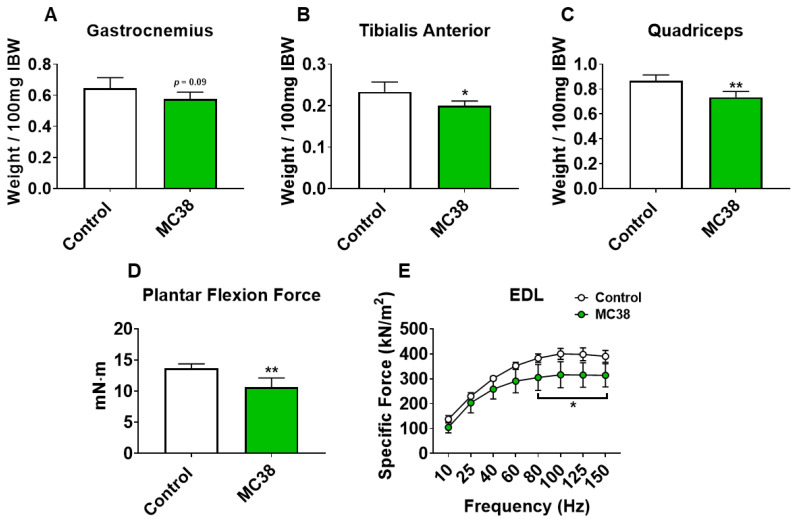
Growth of subcutaneous MC38 tumors causes muscle wasting and weakness. (**A**–**C**) Gastrocnemius (**A**), tibialis anterior (**B**) and quadricep (**C**) weights normalized to initial body weight (IBW) of C57BL/6 male mice (8 weeks) subcutaneously injected with MC38 tumor cells (1.0 × 10^6^ cells/mouse in sterile PBS: MC38) or an equal volume of vehicle (control) (*n* = 5). In vivo plantarflexion force assessment, reported as absolute force (expressed as mN·m) (**D**). Assessment of ex vivo muscle contractility of extensor digitorum longus muscles, reported as specific ex vivo force (expressed as kN/m^2^) (**E**). Data presented as mean ± s.d. * *p* < 0.05, ** *p* < 0.01 vs. control.

To initially examine the effects of MC38 tumor cell growth on the development of cachexia, male C57BL/6 mice were subcutaneously (interscapularly) injected with 1 × 10^6^ MC38 cells. All the mice, control and MC38, were sacrificed 28 days following tumor transplantation. There was no significant difference in initial body weight (Figure 1A) or final body weight (Figure 1B); however, the body weight change over the course of the experiment was significantly different between the groups (*p* < 0.001; Figure 1C). The carcass weights demonstrated a 9% reduction in MC38 compared to control (*p* < 0.05, Figure 1D), while fat was reduced by 69% in the MC38 mice (*p* < 0.01; Figure 1E). The livers were non-significantly larger (+17%; *p* = 0.08; Figure 1F) and the hearts were unchanged (Figure 1G) in the MC38 mice. Additionally, the MC38 mice experienced severe splenomegaly compared to controls (+241%; *p* < 0.0001; Figure 1H). The MC38-tumor-bearing hosts also revealed atrophy of several skeletal muscles compared to control. Though the gastrocnemius muscles were not significantly reduced (−11%; *p* = 0.09), both the tibialis anterior (−14%; *p* < 0.05) and quadricep (−16%; *p* < 0.01) muscles were wasted in the MC38 tumor-bearing hosts compared to control (Figure 2A–C). Interestingly, despite mild, non-significant changes in the gastrocnemius size, the plantarflexion force at 100 Hz was reduced (−22%; *p* < 0.01) in the MC38 tumor hosts compared to control animals (Figure 2D). Consistent with the in vivo loss of muscle force, the ex vivo contractility assessment of the extensor digitorum longus (EDL) revealed force reductions in MC38 hosts beginning at 80 Hz and continuing to 150 Hz, with an average reduction of 20% (*p* < 0.05; Figure 2E).

### 2.2. Growth of Subcutaneous MC38 Tumors Causes Loss of Cancellous and Cortical Bone

**Figure 3:**
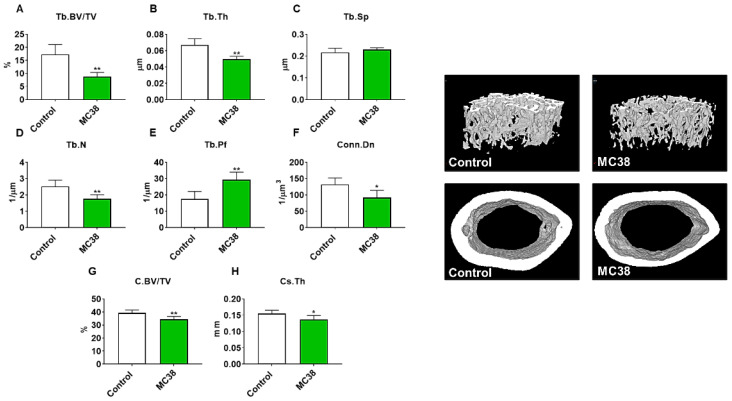
Growth of subcutaneous MC38 tumors causes loss of cancellous and cortical bone. Representative three-dimensional analysis of microcomputed tomography (µCT) scanned images and quantification of trabecular bone volume fraction (Tb.BV/TV) (**A**), trabecular thickness (Tb.Th) (**B**), trabecular separation (Tb.Sp) (**C**), trabecular number (Tb.N) (**D**), trabecular pattern factor (Tb.Pf) (**E**), trabecular connectivity density (Conn.Dn) (**F**), cortical BV/TV (**G**) and cortical cross-sectional thickness (Cs.Th) (**H**) of femurs from C57BL/6 male mice (8 weeks) subcutaneously injected with MC38 tumor cells (1.0 × 10^6^ cells/mouse in sterile PBS: MC38) or an equal volume of vehicle (control) (*n* = 5). Data presented as mean ± s.d. * *p* < 0.05, ** *p* < 0.01 vs. control.

As we have previously demonstrated bone disruptions in models of CRC [12,13,16], we wanted to also assess whether mice bearing subcutaneous MC38 tumors revealed bone alterations. To examine cancellous and cortical bone morphometry, femurs from control and MC38 hosts were analyzed by microcomputed tomography (µCT). Mice bearing subcutaneous MC38 tumors revealed a marked loss of cancellous bone, indicated by reductions in bone volume fractions (Tb.BV/TV; −49%, *p* < 0.01), trabecular thickness (Tb.Th; −26%, *p* < 0.01), trabecular number (Tb.N; −30%, *p* < 0.01) and trabecular connective density (Conn.Dn; −31%, *p* < 0.05), while the trabecular pattern factor (Tb.Pf; +68%, *p* < 0.01) was increased, and trabecular separation was unchanged (Figure 3A–F). Interestingly, the MC38 hosts also revealed loss of cortical bone as indicated by reductions in cortical bone volume fractions (C.BV/TV; −12%; *p* < 0.01) and cortical cross-sectional thicknesses (Cs.Th; −12%, *p* < 0.05) (Figure 3G,H).

### 2.3. Formation of MC38 Liver Metastases Causes Severe Cachexia Consistent with Heightened Skeletal Muscle Wasting and Weakness

**Figure 4:**
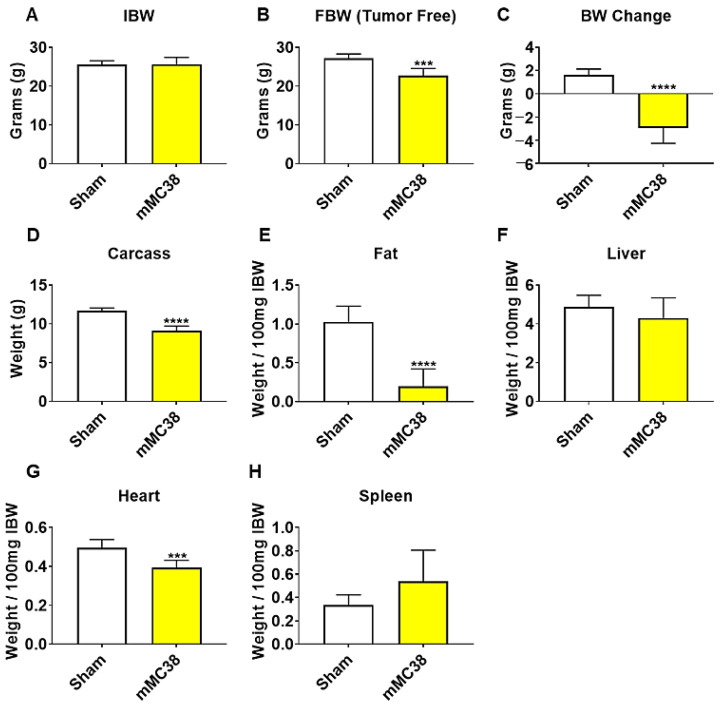
Formation of MC38 liver metastases promotes severe cachexia. (**A**–**D**) Initial body weight (IBW) (**A**), tumor-free final body weight (FBW) (**B**), BW change (**C**) and carcass weights (**D**) of C57BL/6 male mice (8 weeks) intrasplenically injected with MC38 tumor cells (1.25 × 10^5^ cells/mouse in sterile PBS: mMC38) or an equal volume of vehicle (sham) (*n* = 5–8). (**E**–**H**) Fat (**E**), liver (**F**), heart (**G**) and spleen (**H**) weights normalized to IBW. Data presented as mean ± s.d. *** *p* < 0.001, **** *p* < 0.0001 vs. sham.

**Figure 5:**
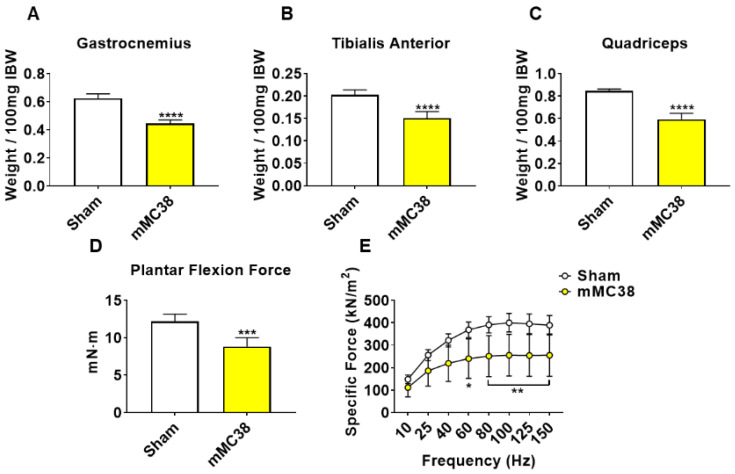
Formation of MC38 liver metastases heightens muscle wasting and weakness. (**A**–**C**) Gastrocnemius (**A**), tibialis anterior (**B**) and quadriceps (**C**) weights normalized to initial body weight (IBW) of C57BL/6 male mice (8 weeks) intrasplenically injected with MC38 tumor cells (1.25 × 10^5^ cells/mouse in sterile PBS: mMC38) or an equal volume of vehicle (sham) (*n* = 5–8). In vivo plantarflexion force assessment reported as absolute force (expressed as mN·m) (**D**). Assessment of ex vivo muscle contractility of EDL muscles, reported as specific ex vivo force (expressed as kN/m^2^) (**E**). Data presented as mean ± s.d. * *p* < 0.05, ** *p* < 0.01, *** *p* < 0.001, **** *p* < 0.0001 vs. sham.

We have previously demonstrated that the formation of LM causes exacerbated cachexia in mice bearing C26 and HCT116 CRCs, and thus wanted to assess whether the formation of MC38 LM similarly presented with a heightened cachectic phenotype [9,12]. To do so, male C57BL/6 mice were intrasplenically injected with 1.25 × 10^5^ MC38 cells. In contrast to the previously described subcutaneous experimental timeline, sham and mMC38 mice were sacrificed 19 days following tumor implantation, as the mMC38 hosts were displaying greatly reduced activity, a hunched over appearance and mild ascites accumulation. There was no difference in initial body weight (Figure 4A); however, the mMC38 hosts displayed reduced final body weights (−17%, *p* < 0.001) compared to the sham-operated animals and a net loss of ~3 g of body weight (*p* < 0.0001) over the 19-day period (Figure 4B,C), consistent with increased cachexia, when compared to the subcutaneous tumor-bearing hosts (Figure 1). Complementing the reduction in body weight, carcass weights were reduced by 22% (*p* < 0.0001), and gonadal fat mass was reduced by 81% (*p* < 0.0001) in the mMC38 hosts compared to sham. Neither the liver nor spleen differed between the groups, while the whole-heart size was reduced by 21% (*p* < 0.001) in mMC38 compared to sham. Similar to our prior findings, and though the experimental sets were conducted at separate times, skeletal muscle wasting and weakness appeared to be exacerbated by the formation of MC38 LM. This was reflected by reduced gastrocnemius (−29%, *p* < 0.0001), tibialis anterior (−26%, *p* < 0.0001) and quadriceps (−30%, *p* < 0.0001) muscles compared to sham animals (Figure 5A–C). Additionally, the plantarflexion force at 100 Hz was reduced by 28% (*p* < 0.001) in the mMC38 tumor hosts compared to the sham animals (Figure 5D), while the ex vivo contractility of the EDL revealed force reductions in the mMC38 hosts beginning at 60 Hz and continuing to 150 Hz, with an average reduction of 35% (60 Hz; *p* < 0.05, 80–150 Hz; *p* < 0.01; Figure 2E).

### 2.4. Formation of MC38 Liver Metastases Causes Loss of Cancellous and Cortical Bone

**Figure 6:**
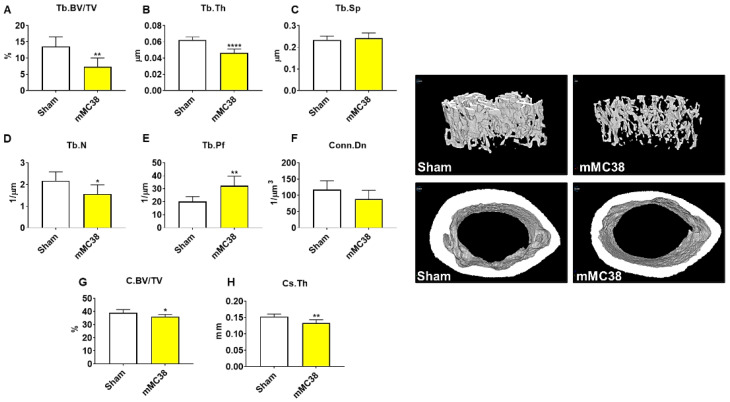
Formation of MC38 liver metastases causes loss of cancellous and cortical bone. Representative three-dimensional analysis of µCT scanned images and quantification of trabecular bone volume fraction (Tb.BV/TV) (**A**), trabecular thickness (Tb.Th) (**B**), trabecular separation (Tb.Sp) (**C**), trabecular number (Tb.N) (**D**), trabecular pattern factor (Tb.Pf) (**E**), trabecular connectivity density (Conn.Dn) (**F**), cortical BV/TV (**G**) and cortical cross-sectional thickness (Cs.Th) (**H**) of femur bones from C57BL/6 male mice (8 weeks) intrasplenically injected with MC38 tumor cells (1.25 × 10^5^ cells/mouse in sterile PBS: mMC38) or an equal volume of vehicle (sham) (*n* = 5–8). Data presented as mean ± s.d. * *p* < 0.05, ** *p* < 0.01, **** *p* < 0.0001 vs. sham.

Our prior work demonstrated that the formation of LM also heightened the loss of bone in experimental C26 CRC, and thus, we also sought to examine whether the formation of MC38 LM aggravated bone loss. Thus, femurs from sham and mMC38 hosts were investigated by µCT. Similar to subcutaneous tumor hosts, mice bearing MC38 LM revealed a marked loss of cancellous bone, indicated by reductions in bone volume fractions (Tb.BV/TV; −46%, *p* < 0.01), trabecular thicknesses (Tb.Th; −26%, *p* < 0.001) and trabecular numbers (Tb.N; −28%, *p* < 0.05), while the trabecular pattern factor was increased (Tb.Pf; +60%, *p* < 0.01) (Figure 6). The trabecular connective density (Conn.Dn) was non-significantly reduced (−25%; *p* = 0.09), while trabecular separation was consistently unchanged (Figure 6). Similarly to the subcutaneous hosts, the mMC38 hosts also revealed a loss of cortical bone as indicated by reductions in cortical bone volume fractions (C.BV/TV; −8%; *p* < 0.05) and cortical cross-sectional thicknesses (Cs.Th; −13%, *p* < 0.01) (Figure 6G,H).

### 2.5. STAT3 Plays a Pivotal Role in MC38-Induced Skeletal Muscle Atrophy

**Figure 7:**
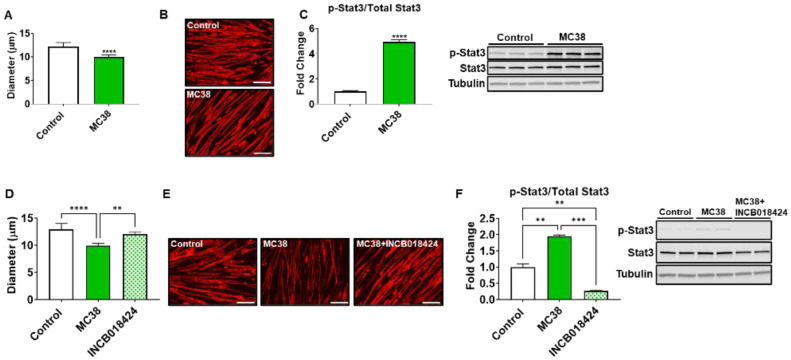
MC38 tumor-derived factors induce atrophy of C2C12 myotubes. Co-culturing of C2C12 myotubes with MC38 tumor cells for 48 h was performed, followed by assessment of myotube diameter (*n* = 400) (**A**). Myotubes were stained with anti-MHC (**B**). Representative Western blotting and protein quantification (fold-change vs. control) for phosphorylated and total STAT3 (*n* = 3) (**C**). Follow-up experiments were performed co-culturing MC38 tumor cells and C2C12 myotubes with or without INCB018424 (400 nM). Quantification of myotube diameter (*n* = 400) (**D**). Myotubes were stained with anti-MHC (**E**). Representative Western blotting and protein quantification (fold-change vs. control) for phosphorylated and total STAT3 (*n* = 2) (**F**). Scale bars: 100 µm. Data presented as mean ± s.d. ** *p* < 0.01, *** *p* < 0.001, **** *p* < 0.001.

We next wished to assess if MC38 tumor-derived factors could directly contribute to skeletal muscle atrophy. Co-culturing experiments revealed an 18% reduction (*p* < 0.0001) in myotubes exposed to MC38 CRC cells, while Western blotting demonstrated a nearly 5-fold increase (*p* < 0.0001) in STAT3 phosphorylation, a signaling pathway strongly implicated in muscle wasting in CRC-induced cachexia (Figure 7A–C) [9,12,13,17,18,19]. To determine if the blockade of the STAT3 axis could preserve myotube size in the presence of MC38 CRC cells, using an approach similar to our previous work [9,17,20], the JAK1/2 inhibitor INCB018424 was added to culture media. INCB018424 was able to rescue the myotube atrophy induced by MC38 cells (+21%, *p* < 0.01), in line with reduced STAT3 phosphorylation (−86% vs. MC38, *p* < 0.001) (Figure 7D–F).

## 3. Discussion

CRC remains amongst the deadliest cancers within the United States and globally, and while advances in treatment and early detection have improved survival rates, the continuous rise in CRC incidence in young adults ensures that this will remain a clinical threat [1,2]. A debilitating byproduct of cancer, cachexia, characterized by the systemic wasting and dysfunction of several organs, including skeletal muscle and bone, occurs in up to 55% of CRC patients [21]. Moreover, the loss of skeletal muscle mass and function associated with cancer-induced cachexia reduces treatment adherence and heightens mortality, while the loss of muscle function can persist in cancer survivors for years [8,21,22,23,24,25]. As cachexia continues to lack viable treatment options, our group has attempted to expand the animal model tool kit to further cachexia research, in particular, for CRC cachexia.

In line with this, recent work from our group has demonstrated that the formation of LM exacerbates cachexia and promotes differential signaling within the skeletal muscle of C26 and HCT116 tumor-bearing mice [9,12]. In the current study, we took a similar approach, utilizing the MC38 CRC cell line, capable of growing in the C57BL/6 background. Similar to our prior findings, the formation of LM appeared to exacerbate skeletal muscle wasting as well as skeletal muscle weakness in mice bearing MC38 tumors (Figure 1, Figure 2, Figure 4 and Figure 5). Though these experiments were not conducted at the same time, therefore representing a limitation of the present study, the markedly reduced muscle wasting (−28%) in animals bearing MC38 LM was considerably higher than that of animals bearing subcutaneous MC38 (−14%) tumors and was achieved in a much shorter time frame (19 vs. 28 days). Altogether, these observations further support the notion that the formation of LM exacerbates muscle wasting and the need to utilize models mimicking a more advanced cachectic phenotype.

Interestingly, the use of the MC38 cell line for the study of cachexia has remained sparse to date, likely due to a lack of evidence supporting the development of cachexia in a subcutaneous context, though several differences between prior investigations and the present study likely account for the discrepancy in findings [15,26,27]. For example, work by Arora et al. [26] and Rohm et al. [27] utilized MC38 CRC in an in vitro context for the study of adipose tissue wasting, reporting non-cachectic effects, whereas in the present study, we report fat wasting in vivo in subcutaneous and LM MC38 tumor-bearing mice (Figure 1E and Figure 4E). Work from Schafer et al. [15] demonstrated an absence of cardiac cachexia in mice bearing subcutaneous MC38 tumors, which is similar to our present findings demonstrating no change in whole-heart size in MC38 subcutaneous tumor hosts (Figure 1G). Notably, mice bearing MC38 LM exhibited whole-heart wasting, similar to our prior findings demonstrating greater reductions in heart size upon LM (Figure 3G) [9,12]. In contrast to the present findings, Schafer et al. [15] reported no loss of skeletal muscle or fat mass. However, this was assessed by EchoMRI, whereas in the present study, we carefully assessed gonadal fat and individual skeletal muscle weights. Additionally similar to Schafer et al. [15], we report no difference in final tumor-free body weight between the control and subcutaneous MC38 hosts (Figure 1). However, our data suggest that the sole assessment of body weight does not account for the severe splenomegaly or the mildly enlarged livers witnessed in the MC38 subcutaneous hosts, which likely contributed to masking the mild cachexia witnessed in our experimental animals. This was reflected by a reduction in carcass weights after all organs were removed (Figure 1D). Additionally, in the present study, 1 × 10^6^ cells were subcutaneously inoculated, while Schafer et al. [15] used 5.0 × 10^5^ cells, which may also account for the differences in findings. Additionally, prior studies did not investigate the direct effect of MC38 tumor cells on muscle atrophy in vitro, whereas we showed marked muscle atrophy in C2C12 myotubes exposed to MC38 cells, consistent with the increased phosphorylation of STAT3, a key mediator of muscle wasting in cancer cachexia [9,12,13,17,20].

As cachexia is characterized by not only a loss of muscle mass but also muscle weakness, to understand whether an animal model properly represents the clinical condition, it is important to also assess muscle function, which was not evaluated in prior studies utilizing MC38 tumor cells. Importantly, despite mild muscle loss in the subcutaneous MC38 hosts, both in vivo plantarflexion assessment and the ex vivo contractility of the EDL muscles revealed a marked loss of muscle function (Figure 1D,E). In further support of exacerbated cachexia upon the formation of LM, both of these parameters were reduced to a greater extent in mMC38 hosts when compared to experimental controls (Figure 5D,E). Regardless, this demonstrates the importance of assessing both muscle mass and function in models of cancer for drawing conclusions regarding cachexia, especially in models where muscle wasting may be mild or non-existent. This is particularly true given the notion that muscle weakness is often sustained in cancer patients following the cessation of cancer treatment, or who are in remission [8,22].

An area of cancer cachexia that has been gaining interest is the multi-organ dysfunction that occurs during disease progression. In particular, we and others have shown that bone loss may accompany muscle loss during cancer cachexia and that a disrupted bone–muscle axis may contribute to disease progression [12,16,28,29,30]. Moreover, we recently demonstrated that the formation of C26 LM not only heightens the wasting of skeletal muscle, but also promotes greater bone loss compared to animals bearing subcutaneous tumors [12,16]. Interestingly, and in contrast to our prior findings, the formation of MC38 LM did not appear to heighten the loss of bone compared to subcutaneous tumor hosts, as both revealed markedly reduced trabecular and cortical bone (Figure 3 and Figure 6). This is likely attributable to the different timelines of the two experimental approaches in the present study. Indeed, the mMC38 hosts were sacrificed 9 days earlier than the subcutaneous hosts, whereas in our published work using C26 cells, both the subcutaneous and LM hosts were sacrificed 2 weeks after tumor implantation. Keeping this in mind, we can only speculate that the formation of LM causes more rapid bone loss in mice bearing MC38 LM, although future studies will need to clarify this matter.

Together, our study identified that MC38 tumors are able to induce musculoskeletal wasting and muscle weakness, with the formation of MC38 LM appearing to worsen cachexia, similar to advanced CRC. Although we did not interrogate any molecular mechanisms responsible for the observed in vivo phenotype, our in vitro findings implicated STAT3 as a mediator of muscle atrophy in response to MC38 cells, in line with our previous observations using different CRC models [9,12,13,17,19]. In addition, here, we also observed a severe loss of whole-heart size in the mMC38 hosts. Given our prior findings that the formation of LM can cause cardiac dysfunction and changes in gene signaling networks, future studies will need to determine if utilizing mice bearing MC38 LM represents a suitable model for the study of cardiac cachexia [13]. Furthermore, the present study was constrained to male mice; hence, additional studies will need to determine whether MC38 tumors, either subcutaneous or metastatic, cause similar alterations in female mice. Lastly, as we showed that anti-cancer drugs can cause a cachexia-like phenotype [29,31,32,33], the inclusion of chemotherapeutics in both subcutaneous and LM models will be required to examine how musculoskeletal wasting is impacted upon MC38 implantation.

## 4. Materials and Methods

### 4.1. Cell Cultures

The murine MC38 cells were a gift of Dr. Xiongbin Lu (Indiana University School of Medicine). The C2C12 skeletal muscle myoblasts were purchased from ATCC (CRL-1772). Both murine cell lines were cultured in high-glucose Dulbecco’s modified Eagle’s medium (DMEM) supplemented with 10% fetal bovine serum (FBS), 1% penicillin/streptomycin (P/S), 1% sodium pyruvate and 2 mM l-glutamine in 5% CO_2_ at 37 °C. Fully confluent (~100%) C2C12 myoblasts were exposed to DMEM containing 2% horse serum, 2 mM l-glutamine and 1% P/S, replacing the media every other day for 5 days to induce myotube formation, as conducted previously [9]. To assess the impact of tumor-derived factors on fiber size, MC38 cells were co-cultured with C2C12 myotubes using transwell permeable inserts (Thermo Fisher Scientific, Waltham, MA, USA #12565009) for 48 h. The JAK1/2 inhibitor INCB018424 (EMD Millipore, Burlington, MA, USA; 400 nM) was added to the culture medium for 48 h in combination with the MC38 co-culture experiments [9].

### 4.2. Animals

The animal experiments were approved by the Institutional Animal Care and Use Committee at the Indiana University School of Medicine. Eight-week-old male C57BL/6 mice (The Jackson Laboratory, Bar Harbor, ME, USA) were group-housed (up to 5 per cage) and randomized into one of the following conditions: the subcutaneous injection of 1.0 × 10^6^ MC38 tumor cells in sterile saline (MC38, *n* = 5) or an isovolumetric subcutaneous injection of vehicle (control, *n* = 5); the intrasplenic injection of 1.25 × 10^5^ MC38 tumor cells in sterile saline (mMC38, *n* = 8) or an isovolumetric intrasplenic injection of vehicle (sham, *n* = 5). The two experiment sets (control vs. MC38; sham vs. mMC38) were conducted at separate times. The procedure to induce LM of MC38 tumor cells was performed as we previously described [9,12,13]. The animals were weighed daily and then euthanized under light isoflurane anesthesia. Following euthanasia, the skeletal muscle, cardiac, fat, spleen and liver tissues were harvested and weighed. The mouse carcasses were weighed, fixed for two days in 10% neutral buffered formalin and then transferred into 70% ethanol, as previously described [13].

### 4.3. In Vivo Muscle Contractility

All the animals underwent in vivo plantarflexion torque assessment the day before sacrifice (Aurora Scientific Aurora, ON, Canada), as previously described [13]. Briefly, the left hind foot was positioned to align with the tibia at 90° and then taped into the footplate force transducer. The knee was clamped at the femoral condyles, and two disposable monopolar electrodes (Natus Neurology, Middleton, WI, USA) were placed subcutaneously posterior/medial to the knee in order to stimulate the tibial nerve. The peak twitch torque was first established in order to determine the maximal stimulus intensity, and mice were exposed to a stimulation of 0.2 ms at 100 Hz to determine the force output.

### 4.4. Ex Vivo Muscle Contractility

The whole muscle contractility of the extensor digitorum longus (EDL) muscles was determined as previously described [33]. EDL muscles were dissected from hind limbs, the tendons were tied to stainless steel hooks using 4–0 silk sutures, and the muscles were mounted between a force transducer (Aurora Scientific, Aurora, ON, Canada). The muscles were then immersed in a stimulation bath with O_2_/CO_2_ (95/5%) and Tyrode solution (121 mM NaCl, 5.0 mM KCl, 1.8 mM CaCl_2_, 0.5 mM MgCl_2_, 0.4 mM NaH_2_PO_4_, 24 mM NaHCO_3_, 0.1 mM EDTA, and 5.5 mM glucose). The muscles were stimulated to contract using supramaximal stimuli between two platinum electrodes. Data were collected via the Dynamic Muscle Control/Data Acquisition (DMC) and Dynamic Muscle Control Data Analysis (DMA) programs (Aurora Scientific). Prior to each contraction bout, the muscle was lengthened to yield the maximum force (L_0_). The force–frequency relationships were determined via an incremental stimulation frequency protocol (0.5 ms pulses at 10, 25, 40, 60, 80, 100, 125 and 150 Hz for 350 ms at the supramaximal voltage) with 1 min rest periods between contractions. The muscle weight and L_0_ were used to determine the specific force.

### 4.5. Microcomputed Tomography Analysis of Femur Bones

Microcomputed tomography (μCT) scanning was performed to assess the morphological parameters of the metaphyseal regions of the femurs, as conducted previously by Bouxsein et al. [34]. Following euthanasia, mouse carcasses were placed in 10% neutral buffered formalin and allowed to fix for 2 days. Following fixation, the carcasses were placed in 70% ethanol, and the right femurs were dissected and prepared for scanning on a high-throughput μCT specimen scanner. All the femur samples were rotated around their longitudinal axes, and images were attained using a Bruker Skyscan 1176 (Bruker, Kontich, Belgium) with an established set of parameters including pixel size  =  9 μm^3^; peak tube potential  =  50 kV; X-ray intensity  =  500 μA; and a 0.3° rotation step. The raw data files were then used to obtain a 3-dimensional cross-sectional image using the SkyScan reconstruction software (NRecon; Bruker, Kontich, Belgium). All the structural measures were calculated on the three-dimensional reconstructed images using the Skyscan CT Analyzer software (CTAn; Bruker, Kontich, Belgium). Trabecular bone was analyzed between 1.0 and 2.0 mm under the femoral distal growth plate using a threshold of 80–255. The trabecular parameters included the bone volume fraction (Tb.BV/TV), thickness (Tb.Th), separation (Tb.Sp), number (Tb.N), pattern factor (Tb.Pf) and connectivity density (Conn.Dn). Cortical bone was analyzed using a threshold of 160–255 in the femoral mid-shaft. The cortical bone parameters included the cortical bone volume fraction (C.BV/TV) and cross-sectional thickness (Cs.Th).

### 4.6. Myotube Diameter

To investigate fiber size, fully differentiated C2C12 myotube cell layers were fixed in ice-cold acetone:methanol (50:50) for ten minutes, blocked for one hour at room temperature and incubated overnight at 4 °C with an anti-Myosin Heavy Chain antibody (MF-20, 1:100; Developmental Studies Hybridoma Bank, Iowa City, IA, USA). The next day, the myotubes were incubated for one hour at room temperature with an AlexaFluor 594-labeled secondary antibody (A11032, 1:500; Invitrogen, Grand Island, NY, USA). Myotube images were captured using an Axio Observer.Z1 motorized microscope (Zeiss, Oberchoken, Germany), and the myotube diameter was assessed at the narrowest portion along the fibers (*n* = 400 fibers per condition) using the ImageJ software [35].

### 4.7. Western Blotting

To obtain protein from the C2C12 myotubes, the entire well surfaces were lysed using RIPAbuffer (150 mM NaCl, 1.0% NP-40, 0.5% sodium deoxycholate, 0.1% SDS, and 50 mM Tris, pH 8.0) with protease (Roche, Indianapolis, IN, USA) and phosphatase (Thermo Scientific, Rockford, IL, USA) inhibitors on ice. The protein extracts were then centrifuged (15 min, 14,000× *g* at 4 °C), and the protein concentration was determined by the BCA protein assay method (Thermo Scientific). Protein samples (20 μg) were separated in 4–15% gradient SDS Criterion TGX precast gels (Bio-Rad, Hercules, CA, USA) at 125 V and, subsequently, transferred to nitrocellulose membranes (30 min at 100 V; Bio-Rad, Hercules, CA, USA). The membranes were blocked at room temperature for one hour with Odyssey blocking buffer (LI-COR Biosciences, Lincoln, NE, USA), and then incubated overnight in primary antibodies at 4 °C. Following the primary antibody incubations, the membranes were washed three times with PBS supplemented with 0.2% Tween-20 (PBST), and then incubated for one hour with either anti-rabbit IgG (H + L) DyLight 800 or anti-mouse IgG (H + L) DyLight 680 secondary antibodies (Cell Signaling Technologies, Danvers, MA, USA). The membranes were again washed three times in PBST, imaged and quantified using the Odyssey Infrared Imaging System (LI-COR Biosciences, Lincoln, NE, USA). The antibodies used were phospho-STAT3 (Tyr705) (#9145) and STAT3 (#12640) from Cell Signaling Technologies (Danvers, MA, USA), and α-Tubulin (#12G10) from the Developmental Studies Hybridoma Bank (Iowa City, IA, USA). Phosphorylated STAT3 levels were normalized to total STAT3, and tubulin was used as a loading control.

### 4.8. Statistics

For the animal experiments, Student’s t-tests were performed to determine differences between control and MC38, and sham and mMC38, unless the variances between groups were found to be significantly different. In this case, a Mann–Whitney nonparametric test was used. In the case of the ex vivo contractility measures, a 2-way repeated-measures analysis of variance (ANOVA) was used, followed by Bonferroni’s post hoc comparisons. For the cell culture experiments, Student’s *t*-tests were used to determine differences between the control MC38 co-cultured cells, while one-way ANOVA tests were performed in the INCB018424 experiments. Post hoc comparisons were accomplished via a Tukey’s test. All the statistical significance was set at *p* ≤ 0.05. All the statistical tests were performed using GraphPad Prism 9.0.0 (GraphPad Software, San Diego, CA, USA), and the data are presented as means ± s.d.

## 5. Conclusions

In conclusion, our findings demonstrate that the growth of subcutaneous MC38 tumors leads to cachexia according to several indices, including skeletal muscle wasting, skeletal muscle weakness and bone loss. Moreover, these data support the notion that the formation of LM exacerbates cachexia in a model of metastatic CRC. Overall, these data support adding the MC38 tumor model into the toolkit for the study of the musculoskeletal deficits associated with CRC.

## Data Availability

Not applicable.
